# A novel computerized functional assessment for human immunodeficiency virus-associated neurocognitive disorder

**DOI:** 10.1007/s13365-013-0195-5

**Published:** 2013-10-01

**Authors:** Liana S. Rosenthal, Richard L. Skolasky, Richard T. Moxley, Heidi Vornbrock Roosa, Ola A. Selnes, Amy Eschman, Justin C. McArthur, Ned Sacktor

**Affiliations:** 1 Department of Neurology, Johns Hopkins University, 600 North Wolfe Street, Baltimore, MD 21287 USA; 2 Department of Orthopaedic Surgery, Johns Hopkins University, 601 North Caroline Street, Baltimore, MD 21287 USA; 3 Psychology Software Tools, Inc, 311 23rd Street Ext, Sharpsburg, PA 15125 USA; 4 Falls Road Pavilion, 10751 Falls Road, Lutherville, MD 21093 USA

**Keywords:** HIV dementia [34], Assessment of cognitive disorders/dementia [38], HIV [144], Neuropsychological assessment [205]

## Abstract

Human immunodeficiency virus (HIV)-associated neurocognitive disorder (HAND) is present in 30–60 % of HIV-positive (HIV+) individuals and can be assessed by neuropsychological testing and level of functional impairment. HAND diagnosis therefore requires accurate assessment of functional impairment. The Computer Assessment of Mild Cognitive Impairment (CAMCI) is a computer-based screening tool that includes performance-based measures of functional impairment. We sought to evaluate the CAMCI as a functional assessment tool in HAND. One hundred fourteen HIV+ patients and 38 HIV-negative (HIV−) patients underwent neuropsychological and CAMCI testing. Cognitive status for HIV+ subjects was classified using the Frascati criteria. HIV+ subjects grouped together and classified by cognitive impairment performed worse than HIV− subjects on several of the CAMCI tasks, including following directions to the supermarket (*p* = 0.05, *p* = 0.03), recalling which items to purchase (*p* = 0.01, *p* = 0.02), and remembering to stop at a supermarket (*p* < 0.01, *p* = 0.01) and the post office (*p* < 0.01, *p* = 0.03). After controlling for hepatitis C status and depression symptomatology, the tasks “following directions to the supermarket” and the “recalling which items to purchase” were no longer significant. The “remembering to run two separate errands” tasks retained their significance (*p* < 0.01 for both tasks). A subset of the CAMCI tasks therefore successfully differentiated HIV+ patients from HIV− individuals. Differences in hepatitis C status and depression symptomatology could account for some of the function assessment differences in the CAMCI. These results suggest the CAMCI could be a useful objective performance-based functional assessment in patients with HIV.

**Electronic supplementary material:**

The online version of this article (doi:10.1007/s13365-013-0195-5) contains supplementary material, which is available to authorized users.

## Introduction

With the advent of combined antiretroviral therapy (cART) in the mid-1990s, life expectancy of patients with human immunodeficiency virus (HIV) has improved, including among those with cognitive impairment. While the phenotype is milder, the prevalence of HIV-associated neurocognitive disorders (HAND) has remained remarkably high and HAND is estimated to occur in 30–60 % of HIV-positive (HIV+) individuals (Grant 2008). HAND includes a wide spectrum of cognitive impairment ranging from a mild, asymptomatic form to those who have severe difficulties with everyday function (McArthur et al. 2010). The disease course is also variable with patients' cognition worsening over time, stabilizing, or even improving, often depending on compliance with cART and possibly on the degree of penetration of the cART across the blood–brain barrier.

Patients with HAND have impairments in everyday functioning. They have increased dependence with instrumental activities of daily living (IADL) (Heaton et al. 2004), are more likely to be unemployed (van Gorp et al. 1999; Benedict et al. 2000), and are more likely to have impaired driving (Marcotte et al. 1999, 2004). They also have lower medication adherence (Hinkin et al. 2002; Andrade et al. 2005), increasing an individual patient's risk for adverse virologic and clinical outcomes (Gifford et al. 2000). Finally, patients with all stages of HAND have an increased mortality risk (Ellis et al. 1997; Power et al. 2009).

The Frascati criteria for diagnosing HAND combines functional and neurocognitive assessments and stratifies individuals into three categories: (1) Patients with asymptomatic neurocognitive impairment (ANI) exhibit impairment on neuropsychological testing in at least two cognitive domains but do not have functional difficulties; (2) Those with mild neurocognitive disorder (MND) also have impairment on neuropsychological testing in at least two domains but demonstrate mild functional difficulties; and (3) Patients with HIV-associated dementia (HAD) exhibit marked impairment on neuropsychological testing and functional impairment that interferes with day-to-day activities (Antinori et al. 2007). Diagnosing a patient with HAND therefore requires an accurate assessment of their functional abilities. Current functional assessments are based principally on self-report or are too time consuming to administer in a research or clinical setting.

The Computer Assessment of Mild Cognitive Impairment (CAMCI)^1^ was designed and validated to test mild cognitive impairment in older, HIV-negative (HIV−) patients (Saxton et al. 2009) and has been modified for use in an HIV+ population. Becker et al. (2011) demonstrated that the CAMCI is sensitive to mild forms of cognitive impairment in an HIV+ population.

This investigation sought to determine if the CAMCI could be used to assess functional impairment in an HIV+ population, thereby allowing its use to diagnose HAND and differentiate cognitive status among HIV+ individuals. We hypothesized that subjects with HAND will perform worse on CAMCI neuropsychological and functional tests compared with HIV− control subjects and that subjects with HAD will perform worse than those with ANI and MND.

## Methods

### Standard protocol approvals, registrations, and patient consents

This study was reviewed and approved by the institutional review board at Johns Hopkins University. Written informed consent was obtained from all patients participating in the study.

### Participants

One hundred fourteen HIV+ individuals and 38 HIV− individuals were seen at the General Clinical Research Clinic at Johns Hopkins Hospital in Baltimore, Maryland from 2007 to 2008. HIV+ patients were recruited from the Infectious Disease (Moore) Clinic for HIV care at Johns Hopkins Hospital. We also identified prospective participants through our other clinical research studies at Johns Hopkins, specifically the Northeast AIDS Dementia cohort (McArthur et al. 2004) and the Oxidative Stress Cohort (Mohamed et al. 2010). Demographically matched HIV− individuals were recruited from inner city Baltimore via flier distributed throughout Johns Hopkins Hospital. In addition, we asked our HIV+ participants if they knew family or friends who were HIV− who would consider participating in the investigation, thus explaining the overall low education level and high frequency of drug abuse. Inclusion criteria were the following: adults 18 years or older, ability to provide written informed consent, and ability to ambulate at first clinic visit. Exclusion criteria were the following: history or current opportunistic central nervous system infection, history or current schizophrenia, current severe affective disorder believed to explain a subject's cognitive impairment, history of a chronic neurological disorder including multiple sclerosis and epilepsy, and current intoxication on illegal drugs or alcohol. Using these exclusion criteria, a small number of cases were excluded.

All participants underwent a clinical assessment in which basic demographic data were obtained as well as a complete medical, psychiatric, and neurologic history. Substance abuse information was ascertained using a self-assessment questionnaire (Valcour et al. 2004). Alcohol binges were defined as five or more drinks within 2–4 h. A neurologic examination was also performed on the HIV+ individuals. Laboratory testing confirmed HIV serostatus. Hepatitis C status was confirmed with laboratory testing for HIV+ individuals and by self-report for HIV− individuals. Laboratory testing for the HIV+ individuals also included CD4 T cell counts and HIV RNA levels in the serum. Urine toxicology was performed at the time of the visit to determine acute intoxication. Seventy-eight HIV+ subjects also underwent a lumbar puncture and subsequent testing of CD4 T cell counts and HIV RNA levels in the cerebrospinal fluid (CSF). Depression symptomatology was tested using the Beck Depression Inventory (Beck and Beamesderfer 1974) and a score greater than 16 was defined as depression. The North American Reading Test was also administered as a measure of premorbid intellectual abilities.

### Classification of cognitive status

Neuropsychological testing was also performed on all subjects. Tests included semantic and verbal fluency tests (McCarthy and Warrington 1990), Timed Gait test (Robertson et al. 2006), Grooved Pegboard test (Klove 1963), Trail Making tests A and B (Reitan and Wolfson 1985), Digit Symbol Substitution test (Wechsler 1981), the Rey Auditory Verbal Learning test (Rey 1941), the Computerized Reaction time portion of the California Computerized Assessment Package (CALCAP) (Miller et al. 1991), the Odd Man Out test (Flowers and Robertson 1985), and the WAIS III Letter–Number Sequencing test (Wechsler 1997). To assess functional status, we completed the Karnofsky functional performance scale for each subject (Karnofsky et al. 1948) and the Function/Quality of Life scale (Stewart and Ware 1993). Based on these neuropsychological and functional tests, HIV+ subjects were classified into HAND category using the Frascati criteria.

### CAMCI testing

The CAMCI collects data in a standardized fashion on a Tablet PC. The testing takes approximately 25 min and score reports are available immediately. The CAMCI has been validated for detection of mild cognitive impairment among non-demented, community-dwelling adults 60 years and older with a sensitivity of 86 % and a specificity of 94 % when compared to adjudication diagnosis based on paper and pencil tests (Saxton et al. 2009). The CAMCI utilized in our study was modified for use in an HIV population. Items removed from the original CAMCI included many of the items that were not scored but rather included to provide additional information to the clinician. Specifically, the removed items include a few of the orientation questions, the self-reported questions regarding depression, anxiety, and alcohol use, and the individual perception of memory questions. The verbal recognition neuropsychiatric test was also removed. Items added to the original CAMCI included more trials in the Simple Reaction Time task and the Digit Span Forward and Reverse tasks.

The modified version of the CAMCI administered to each subject consisted of seven neuropsychological tests and six functional tasks. At the beginning of the CAMCI administration, research staff type in the participants demographic information and start the testing. The approximately 25-min program then runs independently with the participant interacting with the computer with a stylus. The computer asks the participant the date and then begins with self-report questions involving previous computer use and driving. The neuropsychological tests then begin. Directions come up on the screen and are spoken aloud for each test. The tasks include Simple Reaction Time, Recurring Picture, Go/No Go rule 1 and rule 2, Word Recall, and Digit Span Forward and Digit Span Reverse. Together, these tasks assess attention, executive function, different forms of learning and memory, and speed of processing.

The functional task consists of a simulated supermarket–shopping trip and includes following directions to the supermarket (Shopping Trip Directions task) (Video 1), remembering four items to purchase at the market (Shopping List task), and subjects are told that they must remember to stop at the bank (Errand—bank) and post office (Errand—post office) (Video 2) on their way to the supermarket. When the task begins, the image on the screen indicates that they are in a residential neighborhood as seen in first person through the windshield of a car on the road. Written directions to the supermarket are located on the bottom right hand corner of the screen. While driving, they pass a few other cars as well as people on bikes. While following the directions, the sign for the bank will come into view on the left hand side of the road and the subject needs to touch the stylus to the sign in order to stop at the bank. At the bank, they must perform a transaction with the automated teller machine (ATM). After leaving the bank, the post office comes into view on the right hand side of the road and the subject must remember to touch the stylus to the post office sign.

Once at the supermarket, the computer presents to the subject pictures of supermarket items and the subject must select which items they were supposed to purchase (completion of the Shopping List task) (Video 3). Following this assisted recall task, there is an incidental recall task in which the subject is asked to choose from among other pictures what other people or objects they noted during the driving task (for example, the bus that drove past the subject's car).

### Statistical analysis

Subjects were divided according to their HIV serostatus, and chi-squared or analysis of variance (ANOVA) was used to compare performance between HIV− and HIV+ subjects on the neuropsychological and CAMCI tests. Comparisons of each neuropsychological and CAMCI test were also made between HIV− and HIV+ subjects who were classified into HAND status based on the Frascati criteria. Again, chi-squared or ANOVA was used as appropriate. Results were considered significant if the *p* value was ≤0.05. Sensitivity and specificity of each portion of the CAMCI was determined, using the Frascati criteria diagnosis as the gold standard. Regression analysis sought to correlate CAMCI scores with scores on functional measurements. As there were some significant differences between the group demographics, we used a general linear model to fit the CAMCI test as a function of HIV serostatus, adjusting for baseline hepatitis C status, depression symptomatology according to the Beck Depression Inventory, and North American Reading Test (NART) scores. Pairwise comparisons were made using Tukey's test and Dunnett's *T* test. Stata 10.0 software (StataCorp LP, College Station, TX) and SAS (SAS Institute, Cary NC) were used for the statistical analysis.

## Results

### Participants

Table 1 contains the demographic characteristics of the 38 HIV− subjects and 114 HIV+ subjects. The HIV+ subjects are shown as a group and also classified into their HAND diagnosis of normal cognition, ANI, MND, and HAD. When comparing HIV− to HIV+ subjects, there were no differences in age, gender, education, race, history of computer use, history of ATM use, or history of driving, though HIV+ subjects were less likely to be currently driving (*p* = 0.01). The groups were also similar in their alcohol and illicit drug use, with differences noted only in heroin use in the past 6 months (18 % of the HIV− group versus 6 % of the HIV+ group, *p* = 0.02). Finally, HIV− and HIV+ subjects had different rates of hepatitis C infection (27 % of HIV− subjects and 59 % of HIV+ subjects, *p* < 0.001), depression symptomatology (mean Beck Depression Inventory score of 5.5 in HIV− subjects and 10.3 in HIV+ subjects, *p* < 0.00), and NART scores (mean NART score of 100.2 in HIV− subjects and 95.9 in HIV+ subjects, *p* = 0.02). When comparing HIV− subjects and the HIV+ subjects classified into their HAND diagnosis, there were no differences in age, gender, education, race, history of computer use, history of ATM use, history of driving, and current driving rates. The differences in hepatitis C status (*p* = 0.02) and depression rates remained (*p* < 0.01). There was no difference in NART scores between all of the groups (*p* = 0.13).

**Table 1:** Demographic information

	HIV serostatus			HIV+					
	HIV−	HIV+	*p* value	HIV−	NML	ANI	MND	HAD	*p* value
Total (*n*)	38	114		38	16	37	22	39	
Mean age (SD)	44.3 (10.2)	46.8 (6.4)	0.16	44.3 (10.2)	45.4 (6)	45.9 (6.6)	46.8 (6.4)	48.3 (6.3)	0.22
Education, mean (SD)	12.6 (1.8)	12.7 (2.2)	0.81	12.6 (1.8)	12.3 (1.8)	13.0 (2.8)	12.7 (1.3)	12.6 (2.1)	0.86
Male, no. (%)	26 (68.4)	79 (69.3)	0.92	26 (68.4)	12 (75)	27 (73)	12 (54.6)	28 (71.8)	0.58
Black/African-American/Caribbean, no. (%)	32 (84.2)	105 (92.1)	0.16	32 (84.2)	13 (81.3)	34 (91.9)	21 (95.5)	37 (94.9)	0.32
Hepatitis C positive, no. (%)	10 (27.0)	67 (58.8)	0.00	10 (27.0)	8 (50)	21 (56.8)	13 (59.1)	25 (64.1)	0.02
Beck Depression Inventory, score (SD)	5.5 (6.7)	10.25 (9.7)	0.00	5.5 (6.7)	7 (7.1)	9.2 (10)	7.3 (8.4)	14.2 (10)	0.00
NART, mean score (SD)	100.2 (9.7)	95.9 (9.7)	0.02	100.2 (9.7)	98.2 (10.7)	97.0 (11.2)	94.6 (8)	95 (8.8)	0.13
History of computer use, no. (%)	35 (92.1)	97 (85.1)	0.27	35 (92.1)	14 (87.5)	32 (86.5)	20 (90.9)	31 (79.5)	0.54
History of ATM use, no. (%)	37 (97.4)	112 (98.3)	0.74	37 (97.4)	16 (100)	37 (100)	21 (95.5)	38 (97.4)	0.74
History of driving, no. (%)	35 (92.1)	97 (85.1)	0.27	35 (92.1)	13 (81.3)	34 (91.9)	17 (77.3)	33 (84.6)	0.39
Current driving, no. (%)	19 (50.0)	32 (28.1)	0.01	19 (50.0)	5 (31.3)	10 (27)	7 (31.8)	10 (25.6)	0.17
Current alcohol use, no. (%)	7 (18.9)	11 (9.9)	0.13	7 (18.9)	1 (6.3)	3 (8.1)	1 (4.6)	6 (15.4)	0.39
Binge drinking in past 30 days, no. (%)	5 (13.2)	13 (11.4)	0.77	5 (13.2)	1 (6.3)	6 (16.2)	2 (9.1)	4 (10.3)	0.83
Lifelong history of illicit drug use, no. (%)	33 (86.8)	101 (88.6)	0.77	33 (86.8)	14 (87.5)	32 (86.5)	20 (90.9)	35 (89.7)	0.98
Lifelong history of cocaine use, no. (%)	26 (70.3)	85 (74.6)	0.61	26 (70.3)	11 (68.8)	28 (75.7)	19 (86.4)	27 (69.2)	0.6
Lifelong history of heroin use, no. (%)	19 (51.4)	55 (48.3)	0.74	19 (51.4)	9 (56.3)	14 (37.8)	14 (63.6)	18 (46.2)	0.6
Illicit drug use in previous 30 days, no. (%)	10 (26.3)	17 (15)	0.11	10 (26.3)	2 (12.5)	5 (13.9)	3 (13.6)	7 (18)	0.59
Cocaine use in previous 6 months, no. (%)	8 (21.1)	16 (14.3)	0.32	8 (21.1)	2 (13.3)	5 (13.5)	1 (4.6)	8 (21)	0.42
Heroin use in previous 6 months, no. (%)	7 (18.4)	7 (6.2)	0.02	7 (18.4)	1 (6.3)	1 (2.7)	1 (4.6)	4 (10.5)	0.16
CD4 count, mean (SD)	n/a	360.4 (206.2)	n/a	n/a	328.3 (126.7)	353.0 (212.6)	417.9 (264.1)	348 (190.2)	0.52
Detectable viral load, no. (%)	n/a	46 (41.1)	n/a	n/a	8 (50)	17 (47.2)	8 (36.4)	13 (34.2)	0.62
Plasma viral load in 100's, mean (SD)	n/a	33.0 (104.3)	n/a	n/a	3.4 (39.8)	20.6 (35.2)	22.6 (38.7)	71.0 (183.8)	0.43
CSF viral load tested, no. (%)	n/a	78 (68.4)	n/a	n/a	11 (68.8)	27 (77.1)	16 (72.7)	24 (61.5)	0.55
CSF viral load detected, no. (%)	n/a	27 (23.7)	n/a	n/a	3 (18.8)	11 (31.4)	4 (18.2)	9 (23.1)	0.71
CSF viral load in 100's, mean (SD)	n/a	49.7 (12.8)	n/a	n/a	3 (2)	3.9 (8.1)	1.9 (3.5)	9.1 (20.2)	0.67

Demographic data comparing HIV− to HIV + subjects as a group and classified into HAND diagnosis

*NART* National Adult Reading Test, *SD* standard deviation, *ATM* automatic teller machine, *CSF* cerebrospinal fluid, *NML* normal cognition, *ANI* asymptomatic neurocognitive impairment, *MND* minor neurocognitive disorder, *HAD* HIV-associated dementia

Subjects who were HIV+ and hepatitis C positive were older (mean age of 48.2 versus 44.9, *p* < 0.01), had less education (12.2 years of school versus 13.4 years of school, *p* < 0.01), had lower NART scores (93.9 versus 98.8, *p* < 0.01), were more likely to have depressive symptomatology (31.3 versus 10.6 %, *p* < 0.01), and were more likely to be African-American (*p* = 0.03). In addition, among subjects who were HIV+, approximately 4 % of the normal cognition group had depressive symptomatology whereas approximately 58 % of those with HAD had depressive symptomatology (*p* = 0.03).

Among the HIV+ patients, the mean CD4 count was 360.4 (standard deviation 206.2) with a mean plasma viral load of 32,987 copies/ml (standard deviation 104,305 copies/ml). The HIV+ groups, those with normal cognition, ANI, MND, and HAD, did not differ in CD4 count, HIV viral load in the plasma, HIV viral load in the CSF, or in the number of subjects who had obtained complete viral suppression with undetectable viral loads in the plasma and the CSF. Fewer total subjects completed lumbar punctures but there were no differences in the lumbar puncture rate between the HIV+ groups (*p* = 0.55). While there was a trend toward increased serum viral load among those with more advanced HAND, the differences were not significant (*p* = 0.43). In addition, there was no difference in the antiretroviral central nervous system penetration effectiveness (CPE index) (Letendre et al. 2008) comparing all four groups (*p* = 0.88) or in the pairwise comparisons.

### Neuropsychological testing

When comparing HIV− subjects to HIV+ subjects, there were significant differences in tests of reaction time and executive function (Table 2). Specifically, HIV− subjects performed better on the CALCAP, Choice reaction time test (434 versus 471.6 s, *p* = 0.03), and the WAIS III Letter–Number Sequencing Test (10.2 versus 8.9, *p* = 0.03). In contrast, the Rey Auditory Verbal Learning Test recognition score was lower among the HIV− subjects (11.7 versus 13.5, *p* = 0.03), though HAD patients had worse performance than HIV+, normal cognition patients (*p* < 0.01).

**Table 2:** Neuropsychological tasks

	HIV serostatus			HIV+					
	HIV−	HIV+	*p* value	HIV−	NML	ANI	MND	HAD	*p* value
*n*	38	114		38	12	34	20	29	
Grooved Pegboard dominant hand, mean in seconds (SD)	81.9 (21.9)	83.9 (21.5)	0.63	81.9 (21.9)	67.1 (5.2)	79.9 (14.5)	82.9 (17.3)	96.0 (28.1)	<0.01
Grooved Pegboard non-dominant hand, mean time in seconds (SD)	89.9 (25.3)	94.7 (25.0)	0.31	89.9 (25.3)	76.6 (8.4)	93.6 (21.7)	96.0 (23.5)	102.9 (30.3)	0.02
Trail Making Part A, mean (SD)	30.7 (10.9)	29.5 (9.9)	0.52	30.7 (10.9)	22.6 (5.8)	27.8 (9.2)	29.4 (10.0)	34.2 (10.1)	0.01
Trail Making Part B, mean (SD)	94.1 (61.9)	90.5 (51.8)	0.73	94.1 (61.85)	70.9 (57.5)	84.6 (47.4)	93 (56.8)	103.4 (49.6)	0.40
Digit Symbol Test, mean (SD)	50.3 (13.5)	44.6 (10.8)	0.33	50.3 (13.5)	58.5 (13.7)	50.3 (10.5)	49.4 (10.3)	39.5 (14.6)	<0.01
CALCAP, choice reaction time, mean (SD)	434 (64)	471.6 (91.2)	0.03	434 (64)	412 (40)	470 (75)	447 (47)	494 (86)	<0.01
CALCAP, sequential reaction time, mean (SD)	569.8 (100.1)	613.9 (122.7)	0.07	569.8 (100.1)	496.8 (76.7)	617.7 (114.4)	592.9 (93.2)	655.0 (114.7)	<0.01
RAVLT total, mean score (SD)	46.3 (9.3)	47.4 (10.3)	0.56	46.3 (9.3)	52.4 (8.0)	50.8 (9.3)	48.1 (8.8)	41.0 (10.4)	<0.01
RAVLT delayed recall score, mean (SD)	8.8 (3.2)	9.0 (3.1)	0.52	8.8 (3.2)	10.2 (2.6)	10.0 (3.1)	9.7 (3.2)	7.7 (3.4)	0.03
RAVLT recognition score, mean (SD)	11.7 (3.5)	13.5 (1.8)	0.03	11.7 (3.5)	14 (1.29)	13.2 (2.3)	13.6 (2.4)	11.5 (2.9)	<0.01
Odd Man Out, mean (SD)	35.7 (3.6)	35.6 (4.0)	0.96	35.7 (3.6)	36.9 (2.3)	36 (3.4)	36.3 (2.3)	34.3 (5.4)	0.17
WAIS III Letter–Number Sequence Test, mean (SD)	10.2 (3.5)	8.9 (3.0)	0.03	10.2 (3.5)	11.1 (4.6)	9.2 (2.3)	9.1 (2.9)	7.6 (2.3)	<0.01
FAS verbal fluency, mean (SD)	40.3 (11.9)	39.1 (12.3)	0.61	40.3 (11.9)	49.7 (11.4)	39.9 (12.2)	37.1 (9.7)	35.4 (12.2)	0.01
Finger Tap dominant hand, score (SD)	n/a	n/a		n/a	49.2 (8.2)	49.2 (9.3)	43.7 (7.2)	44.0 (7.6)	0.01
Finger Tap non-dominant, score (SD)	n/a	n/a		n/a	44.8 (8.6)	42.8 (7.2)	41.6 (5.7)	38.9 (7.7)	0.06

Neuropsychological testing comparing HIV− subjects to HIV+ subjects as a group and divided into HAND diagnosis

*SD* standard deviation, *SCFT* semantic category fluency test, *RAVLT* Rey Auditory Verbal Learning Test, *WAIS* Wechsler Adult Intelligence Scale, *IHDS* International HIV dementia scale, *HVLT* Hopkins Verbal Learning Test, *FP* false positive, *TP* true positive, *NML* normal cognition, *ANI* asymptomatic neurocognitive impairment, *MND* minor neurocognitive disorder, *HAD* HIV-associated dementia

### CAMCI neuropsychological tests

Three of the neuropsychological tests that are part of the modified CAMCI differentiated between HIV− and HIV+ subjects (Table 3). HIV− subjects performed better than HIV+ subjects on the Forward Digit span task (score of 7.6 versus 6.8, *p* = 0.02), Backward Digit span task (score of 6.2 versus 4.7, *p* < 0.01), and Word Recall task (score of 3.7 versus 2.4, *p* < 0.01). These differences were no longer significant when controlling for the demographic differences of hepatitis C infection and Beck Depression Inventory score. When controlling for the differences in NART scores, the Forward and Backward digit span, as well as Word Recall test, retained their significance (*p* < 0.01 for all three tests).

**Table 3:** Performance on CAMCI tasks

	HIV serostatus			HIV+					
	HIV−	HIV+	*p* value	HIV−	NML	ANI	MND	HAD	*p* value
Total *n*	38	114		38	16	37	22	39	
Simple Reaction Time task, mean score/max score (SD)	31.5/32 (1.7)	31.8/32 (0.5)	0.13	31.5/32 (1.7)	32.0/32 (0.3)	31.9/32 (0.3)	31.6/32 (0.7)	31.7/32 (0.5)	0.37
Reaction time in milliseconds, mean (SD)	548.9 (166.6)	607.4 (137.3)	0.03	548.9 (166.6)	575.1 (135.2)	592.5 (110.2)	618.2 (152.1)	628.8 (152.8)	0.16
Reaction time in milliseconds, median (SD)	520.8 (163.9)	585.4 (136.7)	0.02	520.8 (163.9)	550.3 (132.6)	572.6 (114.6)	593.2 (150.8)	607.6 (149.5)	0.10
Go/No Go, Rule 1, mean score/max score (SD)	9.5/10 (1.0)	9.0/10 (1.9)	0.12	9.5/10 (1.0)	9.7/10 (0.5)	9.0/10 (1.6)	8.7/10 (2.2)	8.8/10 (2.3)	0.22
Go/No Go, Rule 2, mean score/max score (SD)	9.5/10 (0.9)	9.0/10 (2.1)	0.17	9.5/10 (0.9)	9.5/10 (1.0)	9.3/10 (1.5)	8.2/10 (2.7)	9/10 (2.4)	0.09
Recurring Pictures, mean score/max score (SD)	16.7/20 (3.2)	15.6/21 (4.0)	0.13	16.7/20 (3.2)	15.3/21 (4.0)	15.5/21 (4.0)	16.1/21 (4.1)	15.7/21 (4.0)	0.60
Forward Digit Span Score, mean score/max score (SD)	7.6/10 (1.8)	6.8/10 (1.8)	0.02	7.6/10 (1.8)	7.8/10 (1.9)	6.9/10 (1.9)	6.8/10 (1.7)	6.2/10 (1.7)	0.01
Forward Digit Span Size, mean score/max score (SD)	6.3/7 (0.8)	5.8/7 (1.1)	0.01	6.3/7 (0.8)	6.3/7 (1)	5.9/7 (1.1)	6/7 (0.9)	5.4/7 (1.1)	0.00
Backward Digit Span Score, mean score/max score (SD)	6.2/10 (2.4)	4.7/10 (2.2)	0.00	6.2/10 (2.4)	5.7/10 (2.7)	5.1/10 (2.1)	3.9/10 (1.5)	4.4/10 (2.1)	0.00
Backward Digit Span Size, mean score/max score (SD)	4.7/6 (1.2)	3.8/6 (1.3)	0.00	4.7/6 (1.2)	4.4/6 (1.4)	4/6 (1.3)	3.2/6 (1.01)	3.7/6 (1.2)	0.00
Word Recall, mean score/max score (SD)	3.7/5 (1.3)	2.4/5 (1.3)	<0.01	3.7/5 (1.3)	2.5/5 (1.2)	2.8/5 (1.4)	2.4/5 (1.1)	2.1/5 (1.4)	<0.01
Shopping Trip Directions, mean score/max score (SD)	16.1/18 (2.2)	15.1/18 (3.2)	0.05	16.1/18 (2.2)	14.4/18 (4.8)	16.1/18 (2.7)	14.7/18 (2.8)	14.5/18 (2.8)	0.03
Shopping List, mean score/max score (SD)	3.7/4 (0.6)	3.1/4 (1.1)	0.01	3.7/4 (0.6)	3.3/4 (1.1)	3.3/4 (1.1)	3.2/4 (1.1)	2.87/4 (1.17)	0.02
Incidental Recall, mean score/max score (SD)	2.3/5 (1.3)	2.0/5 (0.9)	0.09	2.3/5 (1.3)	2.2/5 (1.0)	1.9/5 (1.0)	1.8/5 (0.7)	2.1/5 (1.0)	0.32
ATM, mean score/max score (SD)	5.8/7 (2.0)	5.2/7 (1.9)	0.12	5.8/7 (2.0)	5.1/7 (2.0)	5.4/7 (1.7)	5.4/7 (1.8)	5.2/7 (2.0)	0.59
Errands—bank, no. (%)	30 (79.0)	53 (46.5)	0.00	30 (79.0)	9 (56.3)	17 (46.0)	10 (46.0)	17 (43.6)	0.01
Errands—post office, no. (%)	30 (79.0)	56 (49.1)	0.00	30 (79.0)	9 (56.3)	17 (46.0)	10 (46.0)	20 (51.3)	0.03

Comparison of HIV− and HIV+ subjects classified into HAND criteria on all CAMCI neuropsychological and functional tasks. Differences were noted between the groups on tasks of Forward and Backward Digit span and Word Recall

*SD* standard deviation, *ATM* automatic teller machine, *NML* normal cognition, *ANI* asymptomatic neurocognitive impairment, *MND* minor neurocognitive disorder, *HAD* HIV-associated dementia

When comparing HIV− subjects and the HIV+ subjects classified into HAND stages, the same neuropsychological tests showed significant differences between the groups. The Forward Digit span score was different among the five groups, averaging 7.6 in HIV− patients compared to only 6.2 in patients with HAD (*p* = 0.01). Pairwise comparisons indicated that the differences between the HIV− and HAD subjects, as well as between the HIV+, normal cognition and HAD subjects, were significant. The Backward Digit span task was also different between the five groups, following the same generally downward trend between the HIV− subjects and the HAD subjects (score of 6.2 versus score of 4.4, *p* < 0.01). Pairwise comparisons indicated a significant difference between the HIV− subjects and the MND and HAD subjects and between the HIV+, normal cognition group and the MND subjects. Word Recall scores were also different between the five groups, decreased from 3.7 in HIV− subjects to 2.1 in HAD subjects (*p* < 0.01). The pairwise comparisons indicated a significant difference between the HIV− subjects and all four categories of HIV+ subjects. When the general linear model controlled for hepatitis C infection rates and depression symptomatology, differences between the five groups for each of these tests were no longer their significant.

### CAMCI functional tasks

**Fig. 1:**
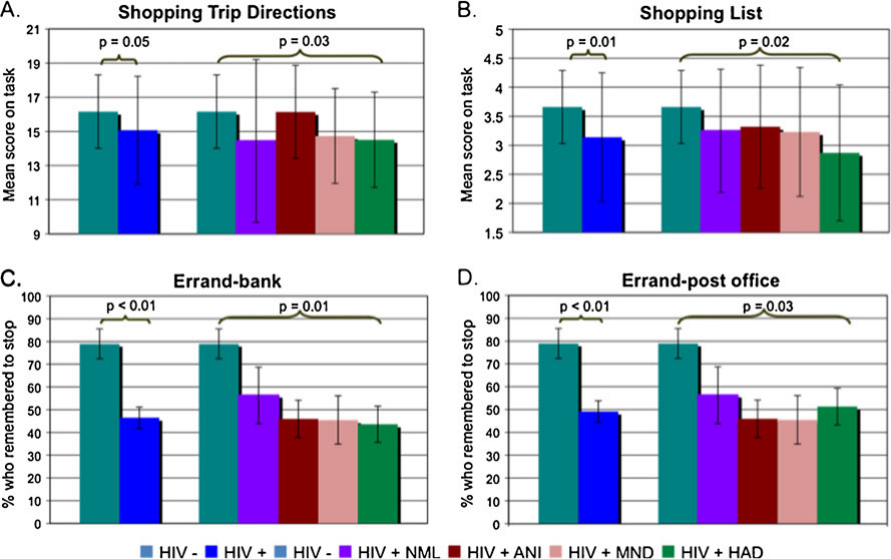
CAMCI Functional Directions. Four of the CAMCI functional tasks demonstrated significant differences in performance between HIV− and HIV+ subjects as well as between HIV− subjects and HIV+ subjects classified into HAND criteria. *NML* = normal cognition; *ANI* = asymptomatic neurocognitive impairment; *MND* = minor neurocognitive disorder; *HAD* = HIV-associated dementia

Four of the functional tasks in the CAMCI were able to distinguish between HIV− and HIV+ subjects (Table 3). Specifically, HIV− subjects performed better on the Shopping Trip Directions task (16.2 versus 15.1, *p* = 0.05), Shopping List task (3.7 versus 3.1, *p* = 0.01), the Errands—bank (79 versus 53 %, *p* < 0.01), and Errands—post office tasks (79 versus 56 %, *p* < 0.01) (Fig. 1). After controlling for hepatitis C status and depression symptomatology, the Shopping Trip Directions task and the Shopping List task were no longer significant. The Errands—bank and Errands—post office tasks retained their significance (*p* < 0.01 for both tasks).

The same functional tasks were significant when comparing HIV− patients and HIV+ patients classified by HAND diagnosis. There was a significant difference between the five groups on the Shopping Trip Directions task (*p* = 0.03) though there were no differences in the pairwise comparisons. The Shopping List task score was different between the five groups, decreasing from HIV− to HIV+ normal cognition to those with HAD (*p* = 0.02). The pairwise comparisons further noted a significant difference between the HIV− subjects and the HAD subjects. Similarly, the subjects' ability to remember to stop at the bank and post office (Errands—bank and Errands—post office) worsened from HIV− to HAD (bank, *p* = 0.01; post office, *p* = 0.03) (Fig. 1). The pairwise comparisons for both these tasks indicated a significant difference between the HIV− subjects and the HIV+ normal cognition subjects. Using the general linear model to control for hepatitis C status and depressive symptomatology when comparing the HIV− subjects to the HIV+ subjects classified into HAND diagnosis resulted in a loss of significance for each of these functional tasks.

Many of the CAMCI functional tasks correlated with conventional measures of functional performance. The Shopping Trip Directions task positively correlated with the Karnofsky performance scale (*r* = 0.19, *p* = 0.04), the Columbia Medication Management Test (*r* = 0.38, *p* < 0.001), and the San Diego Finance test (*r* = 0.30, *p* < 0.001). The Shopping List Task positively correlated with the Karnofsky performance scale (*r* = 0.27, *p* < 0.001) and the Columbia Medical Management Test (*r* = 0.23, *p* = 0.03). In addition, both the Errands—bank and the Errands—post office task positively correlated with the Karnofsky performance scale (*r* = 0.21, *p* = 0.02; *r* = 0.25, *p* = 0.01, respectively) and the Columbia Medication Management Test (*r* = 0.22, *p* = 0.03; *r* = 0.27, *p* = 0.01, respectively).

The area under the curve analysis indicates that forward digit span, forward digit span size, and the functional driving task differentiate between HIV+ individuals with and without HAD. Each of these tests achieved good sensitivity, but relatively poor specificity. For example, a forward digit span raw score cutoff of greater than or equal to 6 gives a sensitivity of 63 % and a specificity of 19 %, a forward span size raw score cutoff of greater than or equal to 5 gives a sensitivity of 80 % and a specificity of 9 %, and a raw score on the Shopping Trip Directions task of greater than or equal to 13 gives a sensitivity of 75 % and a specificity of 18 %.

## Discussion

CAMCI is a computer-based screening tool that includes performance-based measures of functional impairment. Three of the CAMCI neuropsychological tests and four of the CAMCI functional tests differentiated between HIV− and HIV + subjects. The neuropsychological tasks of Forward and Backward Digit span, as well as Word Recall, separated the groups, as did the functional tasks of following directions to the supermarket (Shopping Directions task), remembering four items to purchase (Shopping List task), and remembering to stop at the bank and post office enroute to the supermarket (Errands—bank and Errands—post office). In many of these tasks, the HAD subjects had the worst performance. The CAMCI neuropsychological tests and functional tests also correlated with traditional measures of functional impairment. The CAMCI, therefore, could be a useful objective test of functional performance in HIV+ individuals. We cannot exclude, however, that differences in hepatitis C status, depression, and NART scores contributed to these findings.

With the exception of the CAMCI, the current gold standard of functional assessments are limited by being either time consuming to perform, or by needing to rely on patients' self-report, or both. For example, the Karnofsky performance scale, IADL scale of Lawton and Body, and the Katz/ADL Lawton self-maintenance scale are each assessments that require time and expertise to administer and are based on self-report from either the patient or the provider. Schifitto et al. demonstrated poor correlation between these more self-reported measures of functional impairment and neuropsychological outcomes involving HAND (Schifitto et al. 2001). Other researchers provided objective functional measures through rigorous laboratory assessments including testing abilities to shop, cook, vocational skills, and management of finances and medications (Heaton et al. 1995). While these objective measures were an improvement over subjective functional assessments, such large-scale rigorous laboratory measures could be impractical for both clinical trials and clinical practice.

The CAMCI is a potential alternative to these more cumbersome assessments. We sought to investigate the CAMCI in the HIV population and to compare CAMCI measures in HIV− and HIV+ individuals and in HIV+ individuals with and without HAND, Because there is no biomarker for HAND that can be used as a gold standard, the gold standard for HAND in practice is to use a clinical diagnosis combining neuropsychological test data, functional assessments, and other clinical assessments. These assessments (excluding the CAMCI data) were all used to diagnose HAND from the initiation of the study, so it would not be possible to compare the accuracy of the CAMCI results to the accuracy of the standard neuropsychological tests. Future studies with a limited neuropsychological test battery and functional assessments can be designed from the start to be compared to the CAMCI results.

The sensitivity and specificity of the Shopping Trip Directions task compares favorably to a sensitivity and specificity of 80 and 57 % for the International HIV Dementia Scale (Sacktor et al. 2005). The sensitivity and specificity of CAMCI subtests are also comparable to the sensitivity (71 %) and specificity (46 %) of the Grooved Pegboard non-dominant hand test, an established test for HIV dementia (using a cutoff of 1.5 SD below the age- and education-adjusted mean).

The CAMCI's utility was demonstrated by the correlation between the CAMCI testing and traditional measures of functional impairment as well as the sensitivity and specificity of individual tasks. These measures demonstrate that regardless of hepatitis C status and depression symptomatology the CAMCI tests both correlate with previously validated measures and discriminate between HIV− and HIV+ status. In addition, the CAMCI discriminates between those that are cognitively intact and those that have HAD in a manner that is easy to assess (i.e., a brief computer test).

The CAMCI results also indicate that there may be lower than expected performance in the group that was categorized as HIV+ with normal cognition. The pairwise comparisons are notable for the significant differences between the HIV− subjects and the HIV+ normal cognition subjects on the tasks of Word Recall and remembering to stop at the bank and post office (Errands—bank and Errands—post office). Furthermore, the HIV+ normal cognition group performed poorly on these tasks, with scores closer to subjects with HAND. The HIV+ normal cognition group, therefore, may have subtle cognitive difficulties that are being picked up by these CAMCI tasks. Future research looking at the HIV+ normal cognition group is needed to determine the significance of these results.

This study is limited by the demographic differences between the groups. After controlling for hepatitis C status and depression symptomatology, there were no longer differences between many of the CAMCI scores of the different groups. Subjects with hepatitis C, however, were older and less educated than those without hepatitis C. Older patients and those with less education have both been shown to have poorer performance on cognitive testing and have greater rates of dementia. Therefore, by controlling for hepatitis C serology, we have factored out a potentially large influence on the cognitive test results.

Furthermore, the external validity of our study is challenged by the severity of cognitive disease in our cohort. Many of our cohort patients were recruited because of their concerns and complaints regarding their cognitive status. They therefore do not represent the typical population of individuals with HAND that one would encounter in clinical practice. Later assessments can focus on determining standard scores in a general population and seek to assess the positive predictive value of the CAMCI in a general HIV+ population.

The CAMCI may be a useful objective measure of functional performance in the HIV+ population. While the results are limited by the demographic differences, the CAMCI may be able to assist clinicians in the diagnosis of HAND. This new tool therefore provides an opportunity for closer clinical follow-up and the earlier initiation of treatments specific for HAND.

## Electronic supplementary material

Below is the link to the electronic supplementary material.

Video 1 The video from the Shopping Trip Directions task demonstrates the subject following the directions to the supermarket on the lower right hand corner of the screen. (MPG 2410 kb)

Video 2 The video demonstrates part of the Errands—post office task. After the subject clicks on the Post Office sign, the screen indicates that the car will turn into the post office and then mails a letter. (MPG 2370 kb)

Video 3 The video demonstrates part of the Shopping List task. The subject must remember which item among six choices is on their shopping list given to them at the beginning of the Shopping Trip Directions task. (MPG 494 kb)

## Footnotes

1 Since the conclusion of this study, the product name for CAMCI has been modified to “Computer Assessment of Memory and Cognitive Impairment” to reflect the broad nature of the impairment identified by CAMCI and to stress the importance of the identification of potentially remedial causes.
